# “My Advice is to not let him in”: How Support for Concerned Significant Others is Shaped by Professionals’ Understandings of Alcohol and Other Drug Problems

**DOI:** 10.1177/14550725251388554

**Published:** 2025-10-29

**Authors:** Hans Fagerström, Skogens Lisa, von Greiff Ninive

**Affiliations:** 1 376168 Department of Social work, Stockholm University, Stockholm, Sweden

**Keywords:** AOD problems, critical discourse analysis, CSOs, social services, Sweden

## Abstract

**Aim:**

The study examines how Swedish welfare professionals linguistically construct and legitimize support for concerned significant others (CSOs) of individuals with alcohol and other drug (AOD) problems. This is studied through the professionals’ understandings of AOD problems and how power relations embedded in their language shape the CSO role.

**Methods:**

Semi-structured interviews involving 10 AOD therapists and 10 family care consultants in Sweden were analyzed using Fairclough's critical discourse analysis.

**Results:**

The findings reveal three key logics, in which the CSOs are understood. First, professionals position themselves as educators, aiming to make CSOs understand AOD problems “correctly”. Second, AOD problems are described as an evil force, making CSOs appear passive and reactive. Third, medical and resistance discourses dominate, portraying the AOD problem as an uncontrollable disease, legitimizing strategies such as boundary-setting and self-care. While use of these discourses is argued to reduce stigma and challenge traditional caregiving roles, it also pathologizes CSOs, reinforcing professional authority while limiting CSOs’ perceived capacity for self-determined action and imposing an expectation of self-sufficiency.

**Conclusions:**

The study highlights how medicalization influences CSO support by describing caregiving as dysfunctional, which legitimizes professional intervention while limiting CSOs’ agency. The findings highlight the need for an integrated approach balancing medical and resistance discourses with relational perspectives that emphasize social support.

## Introduction

Concerned significant others (CSOs) of people with alcohol and other drug (AOD) problems are often caught in a difficult position, navigating their relationship with the person having AOD problems. In addition to literature highlighting the emotional, physical and psychological impact on CSOs (D’Aniello et al., 2022; Hellum et al., 2022), studies have also explored support strategies aimed at helping CSOs manage the challenges associated with their position (Di Sarno et al., 2021; Orford, 2017). CSOs play a pivotal role in the lives of people with AOD problems, being both blamed for contributing to AOD problems and credited for facilitating recovery (Orr et al., 2014). Viewing CSOs resources, studies have advocated for their greater involvement in treatment and care (Ventura & Bagley, 2017), including couple- and family-based interventions that equip CSOs with coping strategies to motivate the person with AOD problems towards treatment. At the same time, the influence of CSOs has been used as a rationale to advocate for separate support, based on the understanding that CSOs may contribute to the AOD problem and lack the capacity to control it. Thus, support for CSOs has increasingly emphasized their own needs, aiming to reduce their feelings of responsibility and guilt while fostering autonomy and self-care (Selbekk et al., 2018).

The way professionals describe AOD problems is not a neutral process but rather part of a broader discursive practice. Following Fairclough (1992), discourses are understood here as ways of using language that represent and shape social practices by constructing particular meanings and positioning people and problems in specific ways. In this article, constructions refer to the specific descriptions and interpretations expressed by professionals, which are understood as situated within these broader discourses. As Fairclough argues, language is not merely a tool for communication but a social practice that both reflects and reinforces power relations. In the context of welfare services, this suggests that professionals, through linguistic constructions, contribute to shaping authority and defining both problems and solutions. The contrasting ways in which professionals assess CSOs involvement can be understood as part of broader social constructions that shape assumptions about what AOD problems represent and to what extent they are understood as individual conditions or embedded in social and relational dynamics. The ways in which professionals linguistically construct AOD problems – for example, as a neuropsychiatric disorder within the brain disease model (BDMA) (Volkow et al., 2016), or as a consequence of adverse home environments and parenting styles (Lander et al., 2013) – influences not only the role assigned to CSOs, but also the type of support CSOs receive. However, despite the central role professionals play in shaping both understandings of AOD problems and the positioning of CSOs, there is limited research on how their descriptions of AOD problems influence the support available to CSOs.

Drawing on critical discourse analysis (CDA) as developed by Fairclough (1992), this study aims to enhance how welfare professionals’ discursive understandings of AOD problems shape the support offered to CSOs. By examining the dominant discourses in professionals’ descriptions, the study explores how these discourses position both professionals and CSOs in terms of power and agency and how they legitimize particular support practices. Specifically, the study asks: 1) How do professionals talk about and construct AOD problems in their work with CSOs, 2) How are the CSOs positioned within these constructions, and 3) How do these constructions influence the type of support provided to CSOs?

### Support to CSOs in a Swedish Context

In 2009, Sweden's Social Services Act was clarified to mandate local social services to support CSOs caring for older adults, people with disabilities and those with long-term illnesses, including AOD issues. The aim is to reduce CSOs’ burden and health risks, ensuring they receive support in their own right and are involved in care planning (Ministry of Health and Social Affairs (MHSA), 2022). In addition to means-tested support, such as home assistance and various forms of respite care decided by a care manager at the municipality, psychosocial support for CSOs may be implemented in various ways, and its organization varies across the country. This variation occurs in terms of the type of support (individual and/or group sessions), the specific intervention used and the professional delivering the service (MHSA, 2022). In the context of caring for persons with AOD problems, two main professions are responsible for psychosocial support in Swedish municipalities: AOD therapists and family care consultants (Fagerström, H., Skogens, L., & von Greiff, N., 2025; Winqvist, 2016). AOD therapists specialize in supporting persons with AOD problems^1^ and family care consultants offer a more general approach, supporting not only CSOs of persons with AOD issues, but also other CSOs who provide care. Within the social services organization, family care consultants and AOD therapists are usually separated. AOD therapists operate in the individual and family care services, and family care consultants operate outside this context in “CSO centers” or equivalent settings. Via their role in assessing needs and delivering interventions, these professionals do not merely provide support but also shape understandings of AOD problems and how CSOs are positioned in relation to these problems.

### The CSO's Role in AOD Settings

Support for CSOs has traditionally focused on maintaining their caregiving role rather than addressing their independent needs, reinforcing societal expectations tied to caregiving (Manthorpe et al., 2015; Twigg & Atkin, 1995). This dynamic is particularly relevant in the AOD context, where AOD problems have historically been understood as individual issues, often marginalizing the involvement of CSOs or leading them to be used only as a resource, without acknowledging their own needs, at the same time as blaming them for the AOD problems (Selbekk et al., 2018). For example, Richert et al. (2021) found that Swedish parents experienced conflicting expectations from authorities, being both criticized for overinvolvement, but also expected to support their children when aftercare was lacking and motivate their child toward abstinence and a lifestyle change.

Considering the CSO experience of neglect and disregard from authorities, there has been growing advocacy for relational approaches in AOD treatment settings. While stressing the importance of the CSO's active involvement in the treatment and recovery process, approaches that focus on the relational role of the CSO and their influence on the person with AOD problems have been called for (Selbekk et al., 2018; Tambling et al., 2022). The recovery paradigm is an example of an approach for viewing individual recovery that emphasizes the significant role of the social network, especially close family members (Hennessy et al., 2022). By fostering the individual's relational resources, it is expected that CSOs will take active part in the recovery process. Interventions such as Community Reinforcement and Family Training (CRAFT) exemplify this perspective, equipping CSOs with communications skills and behavioral strategies to positively influence the person with AOD problems by motivating them to enter treatment at the same time as reducing relational stress (Meyers et al., 2002). Other family or couple-based interventions have also demonstrated the value of fostering mutual understanding and improving communication, aiming to repair relational dynamics that might contribute to the maintenance of AOD problems, at the same time as acknowledging the vulnerability of CSOs (Kourgiantakis et al., 2021). These relational views emphasize interdependence within families to a greater extent, with CSO behavior seen as integral to both the AOD problem and recovery. However, challenges persist in implementing these practices because service providers often cite lack of clarity in managing CSO involvement (Lee et al., 2012).

A contrasting, more individually focused approach has also been advocated. Research has shown that support to CSOs usually is explained as only a matter of involvement, without taking the CSO's individual needs into consideration. Even if national policy guidelines explicitly articulate both involvement *and* support in their own right, interventions that primarily focus on the CSO's wellbeing are seldom described (Copello & Templeton, 2012). Yet research has highlighted the need for support aimed at restoring the CSO's sense of identity and agency beyond the caregiving role (Friedrich et al., 2023; McCann & Lubman, 2018). This type of approach encourages CSOs not to try to control or manage their relative's AOD problems because such efforts are viewed as both ineffective and potentially harmful. Instead, strategies such as withdrawal (e.g., establishing independence, engaging in personal activities) are promoted as ways to mitigate the harm caused by caregiving roles (Orford, 2017). By redirecting attention to their own wellbeing, CSOs are supported in moving away from patterns that perpetuate emotional and physical harm. These self-care strategies also aim to alleviate the guilt and self-blame commonly experienced by CSOs, particularly in contexts where parenting styles and attachment theories are cited as contributing to AOD problems (Baumrind, 1991; Lander et al., 2013).

### Family Disease and Codependency

The boundaries between self-care and relational approaches are often fluid, with key concepts such as family disease and codependency exemplifying how these perspectives intertwine. The term “family disease”, which is grounded in family systems theory, highlights the systemic impact of AOD problems, suggesting that each family member adapts in ways that may perpetuate the cycle (Gudžinskienė & Gedminienė, 2011). While this concept emphasizes relational dynamics, it also underscores the need for individual strategies to manage the impact of AOD problems. Viewing the family as a system seeking stability and equilibrium allows an understanding of how CSO involvement applies to AOD problems, illustrating how CSOs may inadvertently sustain addictive patterns in their efforts to maintain balance (Lander et al., 2013). Similarly, codependency, often described as a disease-like state, bridges relational and individual perspectives. It encapsulates dysfunctional caregiving behaviors that exacerbate AOD problems and erode the CSO's autonomy and well-being (Beattie, 1992). While the concept of codependency inherently acknowledges the relational interdependence within families, its frequently calls for individually focused interventions to help CSOs disengage from enabling behaviors and reclaim their agency.

Both the family disease and codependency are terms that are often integrated within the BDMA, which describes AOD problems as a chronic, relapsing brain condition marked by structural and functional changes that influence reward sensitivity, motivation and self-control (Volkow et al., 2022). For CSOs, this biological understanding may provide relief from blame by emphasizing the uncontrollable nature of AOD problems, but it risks neglecting the relational and structural dynamics that shape their experiences. Furthermore, critiques of the BDMA argue that it oversimplifies the multifaceted nature of AOD problems and risks marginalizing the social and environmental factors that significantly impact both individuals and their families (Meurk et al., 2016).

## Methods

### Recruitment and Participants

The participants recruited for this study consisted of AOD therapists and family care consultants from various Swedish social services. The recruitment was based on a previous national survey study where a total of 220 of approximately 290 municipal social services in Sweden participated, which mapped available support for CSOs of people with AOD problems and the professions providing psychosocial support (Fagerström, H., Skogens, L., & von Greiff, N., 2025). It is important to note that AOD therapists and family care consultants could be viewed conceptually as different forms of support, due to their respective organizational positioning inside and outside the AOD context. However, this study viewed both professions as forms of specific support to CSOs in an institutional setting, which could therefore be analyzed as similar professions.

To ensure geographical and municipal diversity (based on population), all 220 social services from the previous study were contacted via central email addresses and/or direct outreach to AOD therapists and family care consultants. Study participation required that professionals regularly provide psychosocial support to adult CSOs of individuals with AOD problems. The recruitment process resulted in 15 family care consultants and 64 AOD therapists agreeing to participate. A further purposive selection was made to ensure variation in municipal size, geographical distribution across the country and at least 1 year of experience providing support to CSOs. A final sample of 20 participants (10 AOD therapists and 10 family care consultants) was deemed sufficient because later interviews confirmed existing findings, indicating that additional interviews would not yield significant new insight. In total, the sample consisted of 17 women and three men. Participants’ experience of working with CSOs of individuals with AOD problems ranged from 4 to 30 years. Most held a university degree in social work, while some family care consultants had alternative professional education in fields such as behavioral sciences, health care or social pedagogy. The majority of participants had also received training in CRAFT and/or twelve-step–inspired programs.

### Interviews

The interviews were conducted using a semi-structured format and carried out via telephone to enable participation from professionals across different regions of Sweden, given the varied organization of social services. Interviews lasted between 40 to 70 min and were recorded and transcribed verbatim. Interview questions focused mainly on how the professionals supported CSOs of persons with AOD problems and why this support is needed. Additionally, the professionals were asked about their views of the trajectory of AOD problems with a special focus on the CSO's role, responsibility, and whether and when the CSO should be involved in treatment alongside the person with AOD problems. The questions also explored organizational dynamics: specifically, how professionals perceived their role in supporting CSOs within social services and how their work related to that of other professionals who also engaged with CSOs.

### Analysis/Approach

The transcripts were coded manually using NVivo (https://lumivero.com/products/nvivo) and the analysis was conducted by the first investigator in regular consultation with the co-investigators to validate and refine emerging interpretations. The analysis was inspired by CDA (Fairclough, 1992), which emphasizes the dialectic interaction between discourse and the surrounding social structures. In this study, linguistic constructions of AOD problems and CSO support were understood as manifestations of broader discourses, with Fairclough's method providing a structured approach to examine how professionals construct support for CSOs based on their understanding of AOD problems and the power dynamics embedded in their language. Moreover, CDA demonstrates how language contributes to the creation of social identities and relationships among people, as well as systems of knowledge and meaning. The analysis is based on Fairclough's three-dimensional model of textual, discursive and sociocultural practices (Fairclough, 1992; see also Jørgensen & Phillips, 2002), where linguistic features were treated not as discourses themselves but as textual indicators used to trace and identify underlying discursive patterns. These patterns were subsequently interpreted in relation to sociocultural practices. The first level primarily focused on modality, transitivity, and nominalization to examine how professionals constructed AOD problems and CSO behaviors. Modality revealed the degree of certainty and how authority was expressed. Transitivity analyzed how agency was assigned (e.g., when AOD problems were described as an evil force acting on CSOs), thereby making the problem, rather than the person, the initiating agent. Nominalization was used to examine how processes or actions were reduced to static concepts, obscuring agency and creating a more abstract representation (e.g., “codependency”) instead of describing the dynamic process of relational entanglement and mutual influence. The second level focused on analyzing the discursive practices professionals used. This involved identifying dominant discourses and examining how these were used to describe AOD problems and approaches to CSO support, suggesting particular ideological stances. Finally, the third level of analysis is presented in the discussion. It contextualizes the professionals’ descriptions within broader sociocultural context and examines how their language reflects and potentially reinforces perspectives on AOD problems and CSOs. By relating these discourses to Swedish social welfare policies, this level of analysis provided insight into how organizational norms, societal expectations and professional responsibilities influence the approaches taken toward supporting CSOs of persons with AOD problems.

### Ethical Considerations

The study was approved by the Swedish Ethical Review Authority (No. 2021-06165-01 & 2022-06087-02). All participants received written and oral information about the study before the interviews and gave their informed consent. Participation was voluntary, and participants were informed that they could withdraw at any time without giving a reason. To ensure confidentiality, all transcripts were anonymized and identifying details were removed.

### Methodological Considerations

Regarding the interviews, efforts were made to clarify to participants that the study focused on professional understandings rather than individual performance. Nevertheless, the sensitivity of these issues may still have influenced how participants chose to present their views. It is also possible that professionals who felt more confident in their perspectives were more inclined to participate, which may have introduced a degree of selection bias. Furthermore, may the use of telephone interviews have limited access to non-verbal cues and somewhat reduced the richness of the data, which should be considered when assessing the findings. Although telephone interviews limit access to non-verbal cues and may yield somewhat less detailed transcripts (Johnson et al., 2021), previous research has shown that they can also enhance perceived anonymity and reduce power dynamics between participant and researcher (Holt, 2010).

## Results

The results are presented in three themes: how professionals construct AOD problems as something CSOs must understand, how these constructions position CSOs as reactive with limited autonomy, and how medical and resistance discourses legitimize particular forms of support. Together, the findings show how such constructions shape the conditions for CSO support within social services.

### Understanding AOD Problems the Right Way

Both AOD therapists and family care consultants stressed the importance of understanding AOD problems. CSOs were portrayed as victims who had tried various strategies to manage or control the person with AOD problems, but these strategies were typically described as dysfunctional. This description justifies an educational and pedagogical approach where the AOD therapists are the experts who aim to help CSOs develop a clear understanding of AOD problems as a phenomenon:

We spend a lot of time describing addiction^2^ – how it arises, what it entails (…) we talk a lot about the power of addiction, that it is a serious, deadly brain disease. (AOD therapist)

(M)y expertise lies in supporting CSOs, providing advice and guidance, informing them about available interventions, perhaps directing them to further resources, offering coaching on how they can relate to their situation. But no, when it comes to – perhaps especially – substance abuse, that is not my area. I am not a therapist; I do not have that competence. (Family care consultant)

The first excerpt above exemplifies a typical stance where AOD problems are explained rather than interpreted. Phrases such as “*how it arises, what it entails*” and “*a serious, deadly brain disease*” demonstrate strong objective modality, leaving little room for alternative perspectives or negotiation and reinforcing the professional's authority as an educator. Further, this statement illustrates how the educational approach shapes the agenda of the support, with the therapist dedicating “*a lot of time*” to explaining the nature of AOD problems. The CSO, meanwhile, is positioned as a participant in a predetermined learning process rather than an active co-creator of meaning. The family care consultant describes their role as advisory and guiding, explicitly states that supporting CSOs dealing with AOD problems requires specialized expertise, which they do not have. In contrast, the family care consultant expresses uncertainty about their role in supporting CSOs dealing with AOD problems, stating “*I am not a therapist*” and emphasizing their lack of specialized competence. This use of uncertain modality legitimizes the division of roles, positioning the AOD therapist as the primary authority in this context.

Even if AOD problems are usually described as a fact, some professionals express less certainty, adopting a more negotiable and situational understanding. The next excerpt from an AOD therapist explains why support for CSOs should remain separate from the person with AOD problems, while acknowledging that joint sessions may sometimes be necessary:

When I have a client (a person with AOD problems) who in practice requests it (inviting a CSO), it is often because they feel that their CSO do not understand the disease. If we see substance abuse as a disease, which I think, many times, you should, then more information is probably the thing I can offer. (AOD therapist)

Although this therapist takes a similar view on AOD problems as a disease, their language reflects a more subjective modality. Phrases such as “*If we see substance abuse as a disease*” and “*which I think, many times, you should*” signal that the therapist incorporates their own perspective, allowing for flexibility and adaptability depending on the context. However, the therapist emphasizes that involving CSOs is not about fostering mutual understanding between them and the client but rather about ensuring CSOs grasp the nature of AOD problems. The therapist and the client are positioned as sharing a common understanding, whereas the CSO is portrayed as lacking this insight. This dynamic legitimizes the therapist's decision to include CSOs in joint sessions: not necessarily to encourage dialogue, but to convince them of what they are “up against”.

This highlights a broader challenge professionals face when engaging CSOs who may resist the dominant understanding of AOD problems. The following excerpt illustrates how an AOD therapist navigates this resistance, emphasizing the need to explain and persuade skeptical CSOs to bridge the gap in understanding:

I think it is a family disease. I see substance abuse, alcoholism, or whatever it may be, as a disease. When you say that, they (CSOs) get irritated – ‘How the hell can you call it a family disease?’ (…) Then you have to explain what you mean by that. So, it's always something. ‘You can’t just blame it on a disease,’ but when you have a disease, you have to take care of it, and it spreads to other people. ‘But I don’t drink, I don’t do drugs.’ No, but this is your behavior; you’ve started lying. It spreads because you are affected by their behavior. (AOD therapist)

This excerpt illustrates how the therapist's personal stance on AOD problems shapes their educational approach to CSOs. The phrase “*I think it is a family disease*” introduces strong subjective modality, suggesting that the entire family is affected and dysfunctional. However, this quickly transitions into stronger objective modalities with statements such as “*it spreads*” and “*yoúve started lying*”, viewing CSO behaviors as consequences of the AOD problem. In addition to reinforcing this conviction, this excerpt demonstrates how the conviction constructs the educational support provided to CSOs. The counterarguments from the CSOs are presented as an expected barrier* – *seen in the phrases “*When you say that*” and *“Then you have to explain what you mean by that*”* – *suggesting a rhetoric that treats skepticism as a routine challenge the AOD therapist must overcome. By asserting AOD issues as a “disease” and a “family disease”, the therapist legitimizes an interpretation of CSO behaviors such as “*lying*” as symptoms of AOD influence, reinforcing the need for professional intervention.

### CSOs as Reactive Rather Than Proactive to AOD Problems

Building on the educational approach and strong modality, this theme examines how AOD problems are constructed as a superior evil force that shapes individuals and relationships. Definitive claims by professionals present the AOD problem as an undeniable fact, laying the foundation for assigning it agency. Transitivity highlights how that agency is assigned linguistically, positioning the AOD problem as the central actor that dictates the dynamics and diminishes the roles of others. CSOs are portrayed as reactive and passive and as being deceived not by the person with AOD problems, but by the AOD problem itself. By constructing the AOD problem as a dominant force, professionals legitimize their role as essential mediators, shaping how CSOs understand their experiences. The following excerpt exemplifies how the AOD problem is constructed as an autonomous force, influencing the person with AOD problems, the CSO and their relationship:

Explaining a bit about what addiction does to a person and like, this brainwashing – how they almost become brainwashed and manipulative, and you could think of it as the person with addiction also manipulating themselves, in a way. Putting it in some kind of context, because often the CSO struggles to understand: ‘He wasn’t like that before. I don’t understand how it could have turned out this way.’ So, really, it's about creating understanding – not as an excuse, but still, an understanding, I think. (AOD therapist)

The phrase “*what addiction does*” constructs the AOD problem as an independent force that brainwashes and manipulates. The nominalization of “*addiction*” becomes apparent as the professional does not describe the *act* of drinking or taking drugs but instead attributes these actions to an external force that has taken control of the person. This construction portrays the AOD problem as stripping the person of agency, transforming them into an unauthentic version of themselves. This reinforces the need for CSOs to understand the unconscious corruption caused by the AOD problem. However, the AOD therapist's clarification “*not as an excuse*” introduces tension: while the explanation aims to reduce blame toward the individual with AOD problems, it simultaneously emphasizes that this understanding should not justify harmful behaviors. Instead, the CSO needs to reconsider relationship dynamics and establish boundaries to delineate what is acceptable. This dual description* – *balancing understanding with accountability* – *illustrates how the perceived agency of AOD problems interacts with CSOs relational responsibilities.

Building on this duality, professionals frequently use the word “*codependency*” to describe CSOs’ entanglement with the AOD problem and how it redefines relationships:

Codependency becomes a broader concept – describing someone affected by another person's substance abuse (…). That's where the term codependency actually comes in, as an enabler, rather than simply being a CSO. (AOD therapist)

It's that, you’re not just a CSO, you can be caught up in codependency. Changing the way you are, being able to focus on yourself. (Family care consultant)

Similarly, to the nominalization of a person's “addiction” or “substance abuse” CSOs are described as becoming or being entangled in “*codependency*”, suggesting that their role as, for example, a parent or partner is reduced or replaced. The concept of codependency reinforces the construction of CSOs as lost agents, with their actions portrayed as reactive rather than self-directed. Efforts to manage the AOD problem, such as lying, lending money, or attempting to control the problem, are described as unintentionally sustaining the very dynamics CSOs seek to escape. Professionals refer to these behaviors as consequences of the AOD problem rather than independent actions. This description not only shifts the perceived source of these behaviors, but also provides an understanding that alleviates guilt and shame, often identified as central struggles for the CSO, by attributing dysfunction solely to the AOD problem itself.

The dominant agency of the AOD problem legitimizes support that encourages CSOs to distance themselves and reclaim their authentic selves. This often necessitates separate support systems to prevent CSOs from being seen solely as resources for the person with AOD problems. However, professionals also describe agency of the AOD problem as fluid, shifting the CSO's position over time.

Sometimes it's like the addiction comes and goes. There are openings where you can work more on, like, reinforcing sobriety and helping the person with addiction to choose. That you can have a goal to help them choose sobriety. (AOD therapist)

The phrase “*comes and goes*” illustrates how professionals navigate the dynamic roles of CSOs, particularly when distinguishing between codependency and opportunities for engagement. As previously discussed, CSOs are described as codependent when they attempt to control the AOD problem, reflecting a reactive agency. However, the transient nature of the AOD problem creates “*openings*” where its dominance weakens, allowing the person with AOD problems to regain some degree of agency.

In these moments, the CSO is positioned as a potential partner who can “*reinforce sobriety*” and *“help them choose sobriety*”. Thus, the temporary retreat of the AOD problem enables the CSO to transition from a reactive, passive, and codependent position to a more engaged and proactive role. This dynamic understanding acknowledges that the CSO's agency is conditional and context-dependent, fluctuating with the perceived presence or absence of the AOD problem's influence. By distinguishing between these states, professionals delineate when the label of codependency applies and when opportunities for relational support emerge, informing their tailored approaches to intervention.

### Balancing Medical and Resistance Discourse

Building on the educational approach of professionals and the portrayal of the AOD problem as an autonomous force, discursive practices emerge through its linguistic constructions. As shown in earlier excerpts, professionals frequently describe the AOD problem as a disease both individual and familial, reinforcing a medical discourse. Within this perspective, codependency is described as a condition with defining characteristics, resembling a diagnosis for CSOs. However, professionals also highlight the limitations of the medical discourse. An overemphasis on codependency risks absolving the person with AOD problems of responsibility, placing an undue burden on the CSO. One family care consultant illustrates this balance:

I think it's really important to communicate that this is a disease (…). You hear a lot of ‘How could he choose drinking over seeing his child?’ And yes, that's absolutely awful. But perhaps it's not a choice in that way; it's a disease. That being said, it doesn’t mean you should treat this person with kid gloves and go along with it just because you pity him for having a disease. That's not the message you want to send. (Family care consultant)

This excerpt illustrates a key limitation of the medical discourse in CSO support: while it reduces stigma by describing AOD problems as a disease, it risks fostering a more caregiving stance that may sustain dysfunctional relationships. The concern that CSOs might “*pity*” or “*go along with it*” reflects how overemphasizing the medical discourse can absolve the person with AOD problems of responsibility, encouraging CSOs to adopt an overly protective role. To mitigate this, professionals integrate a complementary resistance discourse emphasizing boundary-setting and self-care, shifting the focus from caregiving to CSOs’ well-being. Together, these discourses actively balance stigma reduction with empowering CSOs to resist harmful relational patterns. The following excerpt further illustrates the resistance discourse as a core element of CSO support.

I had been in contact with her for a long time, and she asked me, ‘What should I do? My son has come back from the treatment center. He ran away, and now he's in the village where we live.’ She was scared – sometimes she had to drive away from her home because she got afraid when he was intoxicated. She asked me, ‘Should I go to this shrimp dinner with my friends tonight?’ And I said, ‘Yes, I think you should go.’ ‘What should I do if he contacts me?’ ‘Don’t let him in, don’t let him in.’ Of course this is with the understanding that she makes her own choices. You do what you want, but you asked me for advice, and my advice would be don’t let him in. So, she went to the dinner. The son gets a hold of his grandparents, who eventually open the door and let him in. And the result is that he overdoses on his grandfather's couch and dies while his mother is at the shrimp dinner. And I was the one who said, ‘Close the door.’ She still attends my support meetings. He died when he was 26 years old (…). So yes, it's important to be straightforward and clear, and I think that's why people trust me. The mother was away for 60 minutes (at the shrimp dinner) for closing the door. I have a very good relationship with her. I was at the funeral, too. (AOD therapist)

Besides the heartbreaking and nightmarish reality of the CSO seeing her son overdose and die, this excerpt demonstrates how medical and resistance discourses shape CSO support. The phrase “*you asked me for advice, and my advice would be don’t let him in*” exemplifies how boundary-setting and prioritizing the well-being of CSOs become standardized recommendations, stemming from the description of AOD problems as an uncontrollable disease. Expressions such as “*don’t let him in*” and “*close the door*” align with a “*straightforward and clear*” strategy, contrasting with caregiving approaches and legitimizing the idea that the mother's engagement, worry, and efforts to care will not benefit her own well-being. By attending the dinner, the mother enacts resistance, stepping away from a responsibility-driven caregiving role, which is interpreted a fundamental goal in the support. Through this fatal case, the AOD therapist underscores the imperative of self- prioritization and boundary-setting, given that the disease and the actions of the person with the AOD problem remain beyond the CSO's control.

## Discussion

The present study has examined how professionals in the welfare system construct and legitimize support to CSOs through their understanding of AOD problems, revealing three interconnected themes that shape CSO support. The interviewed professionals positioned themselves as educators, emphasizing the importance of creating a shared understanding of AOD problems to help CSOs navigate relationships and the CSO roles. The AOD problem was constructed as an autonomous force with its own agency, resulting in an unhealthy CSO who struggles to prioritize their well-being or maintain functional relationships with the person affected by AOD problems, underscoring the need for professional guidance to help them regain agency and stability. The medical discourse weaves together educational and agency-focused understandings, presenting AOD problems as an uncontrollable disease that also “spreads” to CSOs through concepts such as codependency and family disease. This legitimizes resistance strategies such as boundary-setting and self-care, encouraging CSOs to relinquish caregiving responsibilities and prioritize their own well-being.

Similar to previous studies examining how professionals describe AOD problems, this study confirms the dominance of the medical discourse, even within welfare institutions outside the healthcare sector (Barnett et al., 2018; Volkow et al., 2016). The construction of individual vulnerability remained closely tied to medical terminology, reinforcing the seriousness of AOD problems at the same time as challenging moral assumptions about self-inflicted behavior and weakened character (Frank & Nagel, 2017). A notable observation was how this CSO support, expected to rest on a system-oriented and interpersonal approach, had been reshaped through processes of medicalization, shifting focus toward individualized solutions. Medicalization, “a process by which nonmedical problems become defined and treated as medical problems, usually in terms of illness and disorders” (Conrad, 2007), pathologizes relationships and the act of caregiving by reconstructing them as disorders, or as prone to disorder, through labels such as family disease and codependency. Medicalization reinforces professional authority while deskilling CSOs, making them incapable of making their own health decisions. As caregiving shifts from a relational to an individualized approach, CSOs were described as needing “fixing” through self-regulation and independence. Their ability to pursue interpersonal values, such as belonging and togetherness, became contingent on professional assessments of the AOD problem, reinforcing professional authority while limiting CSOs’ agency and unintentionally undermining their decision-making capacity.

At the same time, resistance discourse by the professionals tended to operate within a complex web of cultural norms. This challenges traditional caregiving roles by problematizing societal expectations, particularly those that often confine parents to caregiving roles without individual agency (D’Aniello et al., 2022). By viewing CSOs through this lens, resistance discourse moves beyond mere interventions to reflect broader narratives about autonomy and self-regulation (Friedrich et al., 2023), which may be empowering, particularly through withdrawal strategies that encourage independence (Orford, 2017). However, such an emphasis also risks conflating independence with self-sufficiency (Weiss, 2002), reinforcing the expectation that individuals must manage their well-being without relying on others. Caring tends to be constructed as an emotional response or as a result of one's role, overlooking its broader relational and interdependent dimensions, including interconnectedness and investment with another individual, idea or project. The concept of good health becomes guided by individual responsibility* – *the individual's responsibility to manage their own body and mind* – *and a “good” CSO is expected to set firm boundaries. Failure to do so is seen as harmful not only to themselves, but also to the person with AOD problems. In this sense, a lack of resistance may reinforce feelings of guilt.

Previous studies suggest that the medical discourse marginalizes CSOs by portraying AOD problems as individual issues, limiting CSO involvement (Selbekk et al., 2018). The present findings indicate that medical discourse, particularly through boundary-setting, serves as a discursive counterbalance to involvement. Rather than being an unintended side effect, the marginalization of CSOs was actively promoted. This contrasts with the recovery paradigm, which emphasizes social networks and relational support (Hennessy et al., 2022). This tension might reflect an ideological struggle between individualistic and more collectivist understandings of recovery, where professionals navigate between fostering social participation and protecting CSOs’ independence. As CSOs themselves have cited arguments in favor of the disease model as counterarguments against its application (Meurk et al., 2016), this model* – *particularly when applied to the individual with AOD problems* – *may limit recovery, deny responsibility and increase stigmatization. For CSOs, this individualistic approach may place excessive emphasis on their own recovery, encouraging withdrawal from supportive roles, which can be seen as taking precedence over relational aspects that might facilitate meaningful change.

### Summary of Findings, Conclusions and Implications

The study shows that the welfare professionals tended to primarily construct AOD problems within a medical discourse, positioning CSOs as reactive or codependent towards the AOD problem itself with limited perceived agency. To counter this, a complementary resistance discourse stressed boundary-setting and self-care, aiming to empower CSOs and challenge caregiving norms. These discourses also reflect a broader ideological struggle between individualized, medicalized approaches that emphasize self-regulation and autonomy and more relational or collective understandings of care, highlighting how authority and support practices are negotiated within welfare contexts.

There is reason to believe that CSOs experiences are not uniform and that they vary depending on such factors as stress levels, available support and coping strategies. Additionally, CSOs’ relationship with and perceived responsibility for the person with AOD problems impact their well-being (Dennis & Champlin, 2021; van Namen et al., 2024). Addressing these diverse needs requires an integrated approach that combines the strengths of the medical discourse* – *such as stigma reduction* – *with the recovery paradigm, which prioritizes relational growth and social connectedness. Developing tools and language to bridge these perspectives could better equip professionals to meet the complex needs of both CSOs and individuals with AOD problems.

This study also suggests a line of reasoning that has yet to be thoroughly examined: namely, whether it is desirable to develop CSO support that is specifically tailored to CSOs supporting individuals with AOD problems. One could argue that this group might benefit from an educational approach that conveys an understanding of AOD problems as a disease to counteract stigma and misconceptions directed at both the individual with AOD problems and the CSOs. However, the present findings suggest a risk that that such an approach could further pathologize CSOs’ experiences and inadvertently increase their sense of blame or responsibility.

## Notes

1. In the Swedish welfare system, treatment for individuals with AOD problems that is inspired by, or strictly follows, the 12-step philosophy is a common psychosocial treatment (Bodin, 2006) and AOD therapists are thus familiar with this perspective.

2. The terms “addiction” [*beroende*] and “substance abuse” [*missbruk*] are both used in Swedish policy and practice, as reflected in the National Board of Health and Welfare's (2019) guidelines, where recommendations are issued for both terms.
